# Human endothelial and foetal femur-derived stem cell co-cultures modulate osteogenesis and angiogenesis

**DOI:** 10.1186/s13287-015-0270-3

**Published:** 2016-01-18

**Authors:** Stefanie Inglis, David Christensen, David I. Wilson, Janos M. Kanczler, Richard O. C. Oreffo

**Affiliations:** Bone & Joint Research Group, Centre for Human Development, Stem Cells and Regeneration, Human Development and Health, Institute of Developmental Sciences, University of Southampton, Southampton, SO16 6YD UK; Human Development and Health, Centre for Human Development, Stem Cells and Regeneration, University of Southampton, Southampton, SO16 6YD UK

**Keywords:** Angiogenesis, Osteogenesis, Endothelial, Foetal, Skeletal

## Abstract

**Background:**

A dynamic vasculature is a prerequisite for bone formation where the interaction of bone cells and endothelial cells is essential for both the development and the healing process of bone. Enhanced understanding of the specific mediators involved in bone cell and endothelial cell interactions offers new avenues for skeletal regenerative applications. This study has investigated the osteogenic and angiogenic potential of co-cultures of human foetal diaphyseal or epiphyseal cells with human umbilical vein endothelial cells (HUVEC) in the presence and absence of vascular endothelial growth factor (VEGF) supplementation.

**Methods:**

Early osteogenic activities of the co-cultures (±VEGF) were assessed by alkaline phosphatase (ALP) activity. Osteogenic and angiogenic gene expression was measured using quantitative polymerase chain reaction. An ex vivo organotypic embryonic chick (E11) femur culture model was used to determine the osteogenic effects of VEGF as determined using micro-computed tomography (μCT) and Alcian blue/Sirius red histochemistry and immunocytochemistry for expression of CD31.

**Results:**

ALP activity and gene expression of *ALP* and *Type-1 collagen* was enhanced in foetal skeletal/HUVECs co-cultures. In foetal diaphyseal/HUVECs co-cultures, VEGF reduced the levels of ALP activity and displayed a negligible effect on *von Willebrand factor (vWF)* and *VEGF* gene expression. In contrast, VEGF supplementation was observed to significantly increase *FLT-1* and *KDR* gene expression in co-cultures with modulation of expression enhanced, compared to VEGF skeletal monocultures. In the organotypic chick model, addition of VEGF significantly enhanced bone formation, which coincided with elevated levels of CD31-positive  cells in the mid-diaphyseal region of the femurs.

**Conclusion:**

These studies demonstrate a differential skeletal response of early foetal skeletal cells, when co-cultured with endothelial cells and the potential of co-culture models for bone repair. The differential effect of VEGF supplementation on markers of angiogenesis and osteogenesis in co-cultures and organ cultures, demonstrate the importance of the intricate temporal coordination of osteogenic and angiogenic processes during bone formation and implications therein for effective approaches to bone regenerative therapies.

**Electronic supplementary material:**

The online version of this article (doi:10.1186/s13287-015-0270-3) contains supplementary material, which is available to authorized users.

## Background

Bone formation and repair are highly orchestrated, temporal coordinated processes involving mechanical, biochemical, molecular and cellular factors. During endochondral bone formation the emergent bone is formed from a cartilage template. As the cartilage matures and remodels, skeletal progenitors form from the incoming vasculature, and differentiate into osteoblasts, which in turn form bone on the cartilage template [1]. A functioning microvasculature is thus critical within the bone regenerative process, providing a supply of oxygen, nutrients and cells, and serve as a conduit for the removal of waste [2]. In the absence of a functional and adequate vasculature network, tissue necrosis and failure of any implanted tissue graft will ultimately occur [3, 4].

There is substantial evidence that the interplay between vascular cells and the cells responsible for bone formation (osteoblasts, osteoprogenitors and skeletal stem cells) is critical in the concomitant development of a functional vasculature and the maintenance of skeletal homeostasis [5, 6]. Indeed, it has been established that the enhancement of the osteogenic differentiation of osteoblasts is due to the direct contact culture with endothelial cells (EC) [7, 8], resulting in an elevation of expression of markers such as alkaline phosphatase (ALP) [9, 10] and type I collagen [11, 12]. Villars and co-workers reported an increase in ALP activity only in contact co-cultures of human umbilical vein endothelial cells (HUVEC) and human bone marrow stromal cells (HBMSC). The gap junction protein Connexin43 (CX43) expressed by both cell types [13–16] was observed to be critical in the coupling of this osteogenic induced mechanism. In contrast, indirect co-culture resulted in decreased ALP activity and increased proliferation of HBMSCs in HUVEC-conditioned media. Moreover, the addition of vascular endothelial growth factor (VEGF) to the culture media had no significant effect on the co-cultures although a negative effect on ALP activity of HBMSC was observed [9].

Kaigler et al. demonstrated that EC-mediated bone morphogenic protein-2 (BMP-2) signalling enhanced HBMSC osteogenic differentiation in co-culture in vitro, with a significant increase in ALP activity. Furthermore, transplantation of co-cultured cells on a polymer scaffold increased bone formation in vivo; however, there was no significant angiogenic response compared to monoculture cell controls [17]. Interestingly, Bouletreau et al. also observed up-regulation of *BMP-2* gene and protein expression by endothelial cells, in response to hypoxia and/or VEGF; however, the authors noted that inhibition of VEGF translation did not abolish this effect, implicating hypoxia as playing a key role in the increase in BMP-2 [18]. Recently, Leszcynska and colleagues demonstrated that direct co-cultures of HBMSCs and HUVECs at distinct ratios (50:50, 80:20 and 20:80) enhanced ALP activity, significantly up-regulating ALP and collagen type 1 gene expression and cell proliferation [12]. Zhang et al. reported that co-cultures of HUVECs and MG-63 osteoblasts result in the proliferation of osteoblasts and elevated levels of collagen type 1 and ALP, and a reduction of osteocalcin, which is a late marker of osteogenesis, close to the mineralisation stage, was also observed [7].

VEGF, a 40-kDa mitogen, has been shown to be a central component in bone development and a prerequisite for a number of processes in bone fracture repair and bone formation. Ferrara and colleagues elegantly demonstrated that the most common isoform VEGF165 and its receptors R1 (FLT-1) and R2 (KDR) are essential for endothelial proliferation, migration, vascular permeability and endothelial cell survival [19]. Chondrogenesis and osteogenesis during endochondral bone formation are dynamically linked with the invasion of vasculature, and VEGF is observed in the hypertrophic chondrocytes as the primary ossification centers form and mineralisation proceeds [20, 21]. VEGF and its receptors have been shown to interact with endothelial cells during bone development as early as E8.5 in mice embryos, with VEGF-R1 (Flt-1) and R2 (Flk-1) knock-outs resulting in lethality due to failure of structural formation of a vascular network [22, 23]. However, less well-known is the interaction of VEGF with skeletal cells such as chondrocytes, osteoblasts and osteoclasts [24]. Street and co-workers demonstrated that a slow release model of VEGF enhances both endochondral and intramembranous ossification whilst inhibition results in a decrease in blood vessel formation, bone formation and callus mineralization [25]. Inhibition of VEGF is also associated with an expansion of the hypertrophic zone and disruption of trabecular bone formation in developing mice femurs [20], however, it has been suggested that the fracture hematoma formed during injury but not during development has potent angiogenic activity through VEGF signalling [26]. Studies on the temporal release of VEGF and dual release of VEGF and BMP-2 from poly-lactic acid scaffolds seeded with HBMC in vivo have shown a significant increase in endochondral bone formation and skeletal defect repair [27, 28].

The current study has examined the interaction of key cell types present during human skeletal development and how exogenous added VEGF affects these processes. Understanding these mechanisms where vascular cells and osteoprogenitor cells combine to induce bone formation, repair and vasculogenesis will enhance approaches to cell-based skeletal tissue engineering.

## Methods

### Materials

Foetal calf serum (FCS) was purchased from Invitrogen Life Technologies, Scotland. Penicillin/streptomycin (Pen/Strep), trypsin/EDTA, minimal essential medium, α-modification (α-MEM) and Medium 199 and other tissue culture reagents were purchased from Lonza, Nottingham, UK. Endothelial cell growth supplement (ECGS) was obtained from Promocell, Heidelberg, Germany. Alkaline phosphatase staining reagents and assay kit and other cell culture reagents were purchased from Sigma Aldrich, Poole, Dorset, UK. Human VEGF-165 was purchased from PeproTech EC Ltd., London, UK. Collagenase B was purchased from Roche Diagnostic Ltd., Lewes, East Sussex, UK. Tissue culture plastics were purchased from Greiner BioOne, okGloucestershire, UK.

### Foetal femur diaphyseal and epiphyseal cell isolation and culture

Human foetal tissues were obtained from female patients undergoing termination of pregnancy in line with the Polkinghome Report guidelines. Informed consent was given in writing by the patients, under ethical approval from Southampton & South West Hampshire Local Research Ethics Committee (LREC 296/100). Femurs from approximately 8 weeks post conception (foot length 5.0–5.5 mm) were isolated from foetuses. The surrounding skeletal connective and muscle tissues were removed from the foetal femur sample and both epiphyses were separated from the diaphysis by micro-dissection (transverse incision through the metaphysis region at either end of the bone collar, when present). Proximal and distal epiphyses were combined in each sample and carefully cut into small segments, as was the diaphysis. The dissected femur parts were submerged in collagenase B in a 6-well plate and incubated overnight at 37 °C, 5 % CO_2_. The cell suspension was centrifuged and re-suspended in α-MEM, supplemented with 10 % FCS and 1 % Pen/Strep. The cells were cultured in a monolayer under standard conditions until 95 % confluency was reached. Culture medium was changed every 3–4 days.

### Human umbilical vein endothelial cell isolation and culture

Human umbilical cords were obtained, following signed consent, from healthy mothers after normal, full-term deliveries at the Princess Anne Hospital, Southampton under ethical approval from Southampton & South West Hampshire Local Research Ethics Committee (LREC 05/Q1702/102). HUVEC were isolated and cultured as described by Jaffe et al. (1973) [29] with minor modifications. The umbilical cord veins were flushed through with 1 × PBS to remove cord blood and drained of any excess fluid. Cords were then infused with a 5 mg/ml solution of collagenase B (Roche) and incubated for 1 hour at room temperature to detach the endothelial lining cells. The collagenase solution was drained from the umbilical cord using a 20 ml syringe and collected in a sterile 50 ml conical tube. The cell suspension was diluted by adding equal amounts of 1 × PBS and then centrifuged at 1000 g for 5 minutes. The HUVEC cell pellet was re-suspended and cultured in endothelial culture medium (Medium 199 (Lonza) supplemented with 1 % Pen/Strep, 10 % FCS, ECGM 0.4 % (v/v) (Promocell) and replenished every 3–4 days.

### Two-dimensional co-culture of diaphyseal/epiphyseal foetal femur cells and human umbilical vein endothelial cells

Foetal femur cells derived from the diaphyseal, epiphyseal regions and endothelial cells were trypsinised prior to confluency, washed and re-suspended in 10 ml of a 1:1 mixture of endothelial culture medium (supplemented with ECGS) and alpha-medium, both supplemented with 10 % FCS and 1 % Pen/Strep. A cell count was performed using a haemocytometer and cell suspension volumes containing 2 × 10^5^ cells were transferred to universal tubes for the different cell types: 8 ml of the above culture medium mixture was added to each tube as basal medium and supplemented with or without 100 ng/ml VEGF. The cell suspension of each tube was transferred at 2 ml/well to a 6-well tissue culture plate and cultured at 37 °C, 5 % CO_2_ for 7 days. A media change was performed on day 4.

### Biochemical analysis

#### Alkaline phosphatase staining

Alkaline phosphatase activity from cells in mono- and co-cultures in 6-well tissue culture plates was measured by washing in 1 × PBS, fixing cells in 90 % ethanol and applying 600 μl of 4 % (v/v) Naphthol AS-MX phosphate (Sigma) and 0.0024 % fast violet B-salt (Sigma) mixed in distilled water. Cells were incubated at 37 °C for up to 40 minutes, when the reaction was stopped and the images captured and processed using a Zeiss Axiovert 200 inverted microscope, software version 4.7.

#### Alkaline phosphatase activity

Alkaline phosphatase activity was measured using a colorimetric assay (P-nitrophenol phosphate (pNPP) turnover) measuring absorbance at 410 nm on an ELx800 spectrophotometer. In brief, 10 μl of cell lysate was transferred to a 96-well clear assay plate and made up to 100 ul with 90 μl phosphatase substrate (Sigma) in 1.5 M alkaline buffer solution (Sigma). The cell lysate was incubated at 37 °C for up to 40 minutes and terminated with 100 μl of 1 M sodium hydroxide prior to reading on the spectrophotometer. Results were expressed as nmol pNPP/h.

### Molecular biology

#### Quantitative RT-PCR analysis

RNA isolation was performed using Qiagen RNeasy Mini Kit. Cultured cells were washed twice in 1 × PBS and lysed using RLT buffer (Qiagen). One volume of 70 % Ethanol was added in order to precipitate the RNA. This was then transferred to an RNeasy spin column and centrifuged to separate the RNA. RNA was eluted from the spin column with RNase-free H_2_O. The SuperScript® VILO™ (Invitrogen 11754050) cDNA synthesis kit was used for cDNA synthesis according to the manufacturer’s protocol; 5X VILO™ reaction mix and 10X SuperScript© enzyme were added to the appropriate amount of RNA and incubated at 25 °C for 10 minutes followed by 2 h at 42 °C. The reaction was terminated at 85 °C for 5 minutes. The cDNA sample was diluted 1:2 with RNase free H_2_O and stored at –20 °C or used immediately for quantitative RT-PCR analysis.

Quantitative PCR was performed using Power SYBR-Green PCR master mix (Invitrogen Life Technology). The reaction was made up with 1 μl of cDNA sample, 12.5 μl of SYBR-Green master mix, 6.5 μl RNase free H_2_O, 2.5 μl of reverse and forward primers for the gene of interest (Table 1). The final reaction mix was transferred to a 96-well plate, centrifuged briefly and analysed using Applied Biosystems® 7500 Real Time PCR system (Life Technology). The resulting data were analyzed using AB7500 SDS Software, version 2.0.5 programme. Cycle threshold (Ct) values for each sample were normalised to the housekeeping gene β-Actin. Fold-expression levels of each target gene were calculated using the ∆∆ Ct method. Sample variation in combining averages of relative gene expression produced strong deviations. In order to overcome this sample variability, we analysed repeat data for relative expression from three patients individually for all genes investigated.

**Table 1:** Quantitative polymerase chain reaction primers of osteogenic and angiogenic genes, with housekeeping gene β-Actin

Gene	Abbreviations	Forward 5'-3'	Reverse 3'-5’
*Human β-Actin*	*Actin*	ggcatcctcaccctgaagta	aggtgtggtgccagattttc
*Human alkaline phosphatase*	*ALP*	ggaactcctgacccttgacc	tcctgttcagctcgtactgc
*Human type I collagen*	*Col1*	gagtgctgtcccgtctgc	tttcttggtcggtgggtg
*Human von Willebrand factor*	*vWF*	gttcgtcctggaaggatcgg	cactgacacctgagtgagac
*Human VEGF165*	*VEGF*	tatgcggatcaaacctcacca	cacagggatttttcttgtcttgct
*Human Flt-1 (VEGF-R1)*	*Flt-1*	aaaggcacccagcacatcat	ttcccccctgcattgga
*Human KDR (VEGF-R2)*	*KDR*	attcctcccccgcatca	gctcgttggcgcactctt

### Organotypic embryonic chick femur culture

Femurs were dissected from 11-day-old chick embryos *(Gallus domesticus).* Soft tissue, including adherent muscles and ligaments, were carefully removed while preserving the periosteum. Non-cultured control femurs were immediately fixed in 4 % paraformaldehyde (PFA). The dissected femurs for organotypic cultures were washed in 1 × PBS and placed in organotypic culture as previously described [30, 31] . The bones were transferred to 6-well plates and positioned on 0.40-μm filter well inserts at the interface between the air and the basal culture medium (1 ml of basal tissue culture medium (TCM) consisting of α-MEM, penicillin (100 U/ml), streptomycin (100 μg/ml), and ascorbic acid 2-phosphate (100 μM) (Sigma). Cultures were maintained in basal medium for 24 h at 37 °C in humidified air with 5 % CO_2_. The organotypic cultures were then incubated in different culture conditions - basal TCM (1 ml); basal TCM + 25 ng/ml VEGF (1 ml) and basal TCM + 100 ng/ml VEGF (1 ml). Culture media was changed daily for the duration of the experiment (10 days) (n = 4 femurs per group). After 10 days culture, the femurs were washed in 1 × PBS (×3) and fixed overnight in 4 % PFA.

### Micro-computed tomography (μCT)

Quantitative 3D analysis of the fixed chick femurs was performed using a SkyScan 1176 scanning system (Bruker μCT, Kontich). Samples were scanned at 18 μm resolution and reconstructed using NRecon software interface (v.1.6.4.6, Bruker μCT, Kontich). Reconstructed femurs were analysed using CT Analyser (v.1.13.2.1+, Bruker μCT, Kontich). Bone volume (BV), tissue volume (TV), bone volume/tissue volume (BV/TV) ratio; bone surface/volume ratio (BS/BV), trabecular number (Tb.N), trabecular spacing (Tb.Sp) and trabecular thickness (Tb.Th) of the femurs were calculated (n = 4).

### Histology and immunohistochemistry

#### Histology

Once analysed by μCT, samples were dehydrated through a series of methanol washes (50 %, 90 % and 100 % in dH_2_O) and incubated in Histo-Clear (National Diagnostics). Following incubation in paraffin wax for 1 h at 60 °C, samples were embedded in wax blocks using an automated Shandon Citadel 2000. Consecutive 7-μm-thick sections were cut throughout the depth of the central femur. Mounted sections were rehydrated through Histo-Clear, graded methanols and dH_2_O before staining with Weigert’s haematoxylin and Alcian blue/Sirius red (A/S), indicators of proteoglycan and collagen deposition respectively. Sections were then dehydrated and mounted with DPX (distyrene plasticizer xylene) before imaging with an Olympus BX-51/22 dotSlide digital virtual microscope using OlyVIA 2.1 software (Olympus Soft Imaging Solutions, GmBH).

#### Immunohistochemistry

Mounted sections were rehydrated through Histo-Clear, graded methanol and dH_2_O washes before incubation with 3 % hydrogen peroxide to quench endogenous peroxidase activity. Sections were then treated with hyaluronidase and 0.5 % Triton-X for cell permeabilisation before incubation with blocking buffer (1 % BSA in PBS) for 1 h at room temperature. Primary antibody solution (CD31 (PECAM1) Source BioScience LifeSciences, 1:100 with blocking buffer) was then added and slides were left overnight at 4 °C. Biotin-conjugated secondary antibody solution (1:100 with blocking buffer) was added for 1 h at room temperature. Biotinylated sections were then incubated with ExtrAvidin peroxidase (1 h at room temperature) and subsequently 3-amino-9-ethylcarbazole (AEC) substrate solution (maximum 10 minutes at room temperature) to visualise positive labelling by generation of brown immune complex reaction product. Light green and Alcian blue were used as counterstains, and slides were mounted with Hydromount (Fisher Scientific, UK). Negative controls included primary antibody exclusion. Images were captured with an Olympus BX-51/22 dotSlide digital virtual microscope using OlyVIA 2.1 software (Olympus Soft Imaging Solutions, GmBH) as described above.

### Statistical analysis

Quantitative polymerase chain reaction data and alkaline phosphatase biochemical activity were analysed using one-way analysis of variance (ANOVA) with Tukey-Kramer multiple comparison post-hoc test, and confirmed using two-tailed unpaired Student’s *t* test on GraphPad Prism 6 software. *P* values ≤0.05 were considered significant. Statistical analysis of μCT data were calculated as mean ± standard deviation. Differences among groups were determined by one-way ANOVA with post-hoc Dunnett’s test and statistical differences were considered to be significant if *p* ≤0.05.

## Results

### Alkaline phosphatase activity and gene expression in co-cultures of EC and FFDSCs

**Fig. 1:**
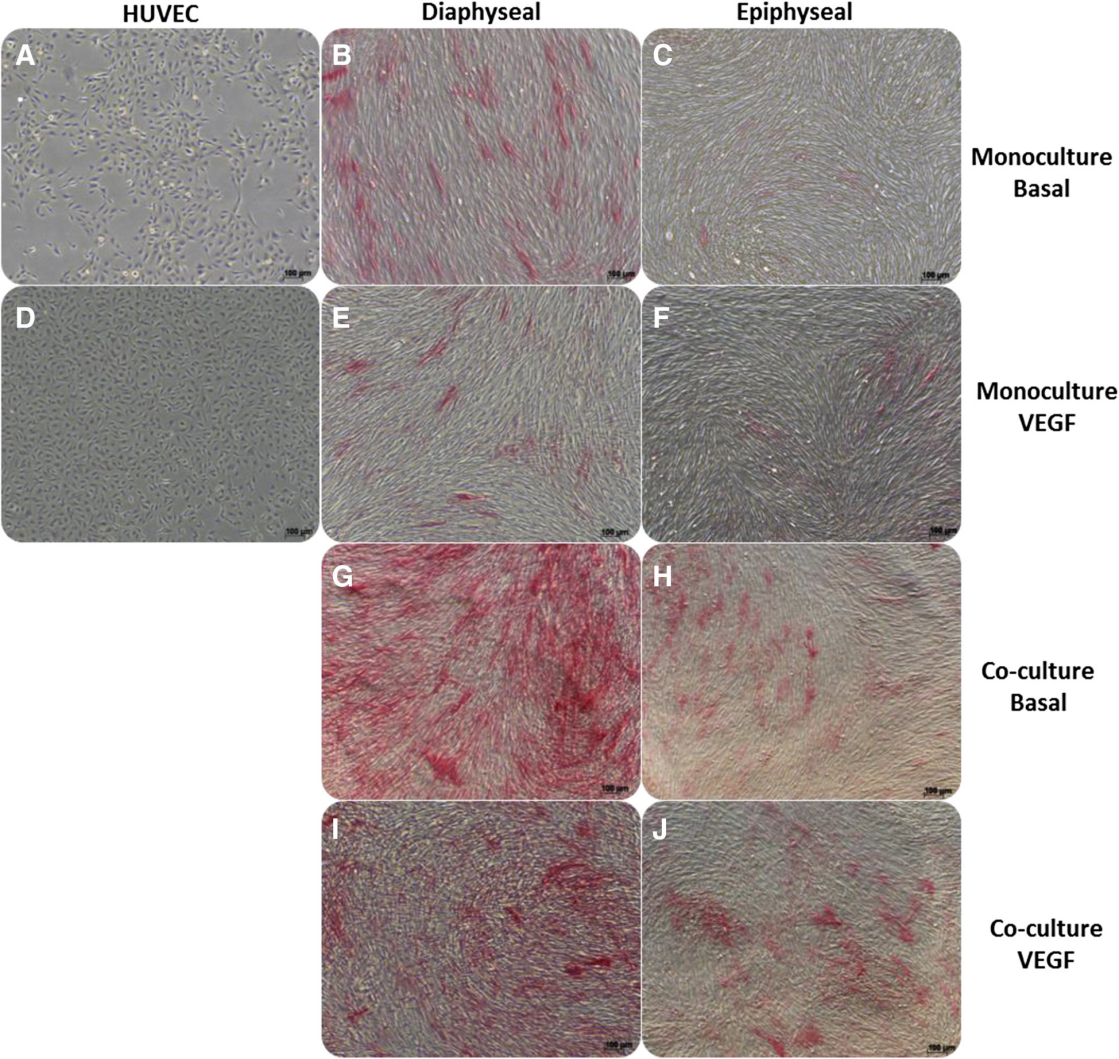
Alkaline phosphatase staining at day 7. Representative images of basal monoculture controls (**a**, **b**, **c**) and monocultures supplemented with 100 ng/ml vascular endothelial growth factor (*VEGF*) (**d**, **e**, **f**) of human umbilical vein endothelial cells (*HUVEC*) (**a**, **d**), foetal diaphyseal cells (**b**, **e**) and foetal epiphyseal cells (**c**, **f**); co-cultures of diaphyseal/HUVEC in basal media (**g**) and supplemented with 100 ng VEGF (**i**); co-cultures of epiphyseal/HUVEC in basal media (**h**) and supplemented with 100 ng VEGF (**j**). *Scale bar* 100 μm

Cell populations derived from the diaphysis of the foetal femur in mono-/co-culture demonstrated enhanced ALP activity in contrast to epiphyseal foetal femur-derived cells or HUVEC after 7 days of culture (Fig. 1). Enhanced expression of ALP was observed in co-cultures of diaphyseal and epiphyseal cells together with HUVEC in contrast to mono-culture populations alone. The addition of VEGF (100 ng/ml) to the cultures reduced the levels of ALP expression (Fig. 1).

**Fig. 2:**
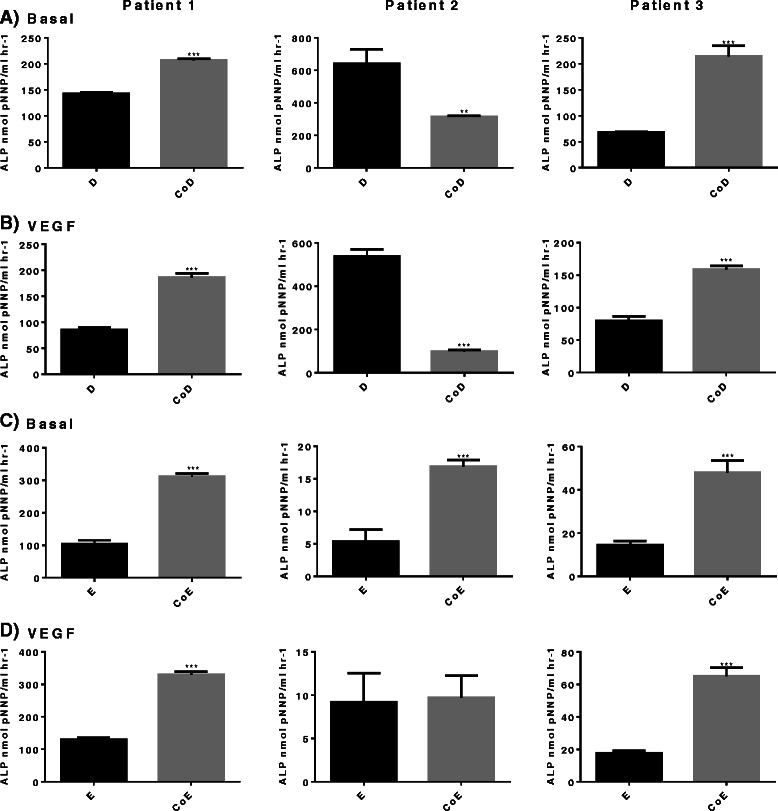
Alkaline phosphatase (*ALP*) activity. ALP activity measured in nmol P-nitrophenol phosphate (*pNNP*)/ml h-1 mono-/co-cultures supplemented with and without vascular endothelial growth factor (*VEGF*) (100 ng/ml). Assays from three separate patient samples. **a** Basal diaphyseal/human umbilical vein endothelial cell (HUVEC) mono-/co-cultures; **b** diaphyseal/HUVEC mono-/co-cultures supplemented with VEGF; **c** basal epiphyseal/HUVEC mono-/co-cultures; **d** epiphyseal/HUVEC mono-/co-cultures supplemented with VEGF. Results are expressed as mean ± SD: *p <0.05, **p <0.01, ***p <0.001. *CoD* diaphyseal co-culture, *CoE* epiphyseal co-culture

Diaphyseal cells (D) in basal mono-cultures displayed enhanced ALP activity, compared to epiphyseal cells (E) in basal mono-cultures, while HUVECs displayed negligible ALP activity (Fig. 2a, c). In basal co-cultures ALP activity was significantly increased in epiphyseal co-cultures (CoE) (Fig. 2c), with approximately three-fold increase in all three patient samples, compared to diaphyseal co-cultures (CoD) (approximately one- to three-fold) in two out of three patient samples (Fig. 2a). Diaphyseal co-cultures also showed a significant approximately two-fold reduction in ALP activity over respective monocultures, in one out of the three patient samples. The addition of VEGF to the cultures resulted in a significant diminution in ALP activity in diaphyseal co-cultures compared to respective basal co-cultures in all three samples (Fig. 2b). In the epiphyseal co-cultures, no significant change was observed in responses to VEGF (Fig. 2d).

**Fig. 3:**
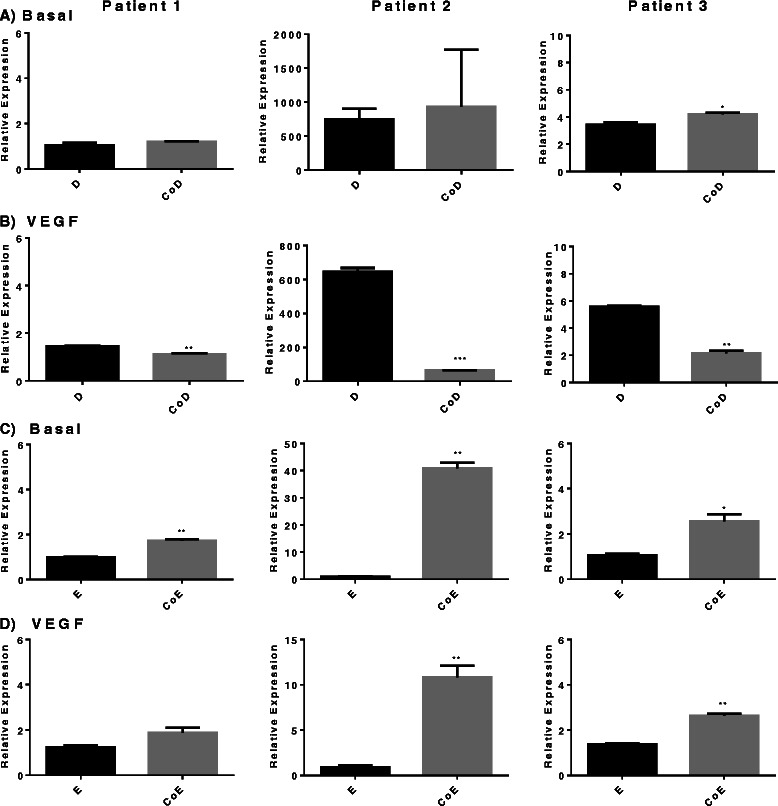
Alkaline phosphatase relative gene expression. Comparison in mono-/co-cultures supplemented with or without vascular endothelial growth factor (*VEGF*) in three patient samples. **a** Basal diaphyseal/human umbilical vein endothelial cell (HUVEC) mono-/co-cultures; **b** diaphyseal/HUVEC mono-/co-cultures supplemented with VEGF; **c** basal epiphyseal/HUVEC mono-/co-cultures; **d** epiphyseal/HUVEC mono-/co-cultures supplemented with VEGF. Results are expressed as mean ± SD: *p ≤0.05, **p ≤0.01, ***p ≤0.001. *CoD* diaphyseal co-culture, *CoE* epiphyseal co-culture

*ALP* gene expression in basal CoD had a varied response to co-culture conditions between patient samples (Fig. 3a), however basal CoE had consistently significantly increased gene expression in all three experiments (range approximately 3-fold to 40-fold difference) (Fig. 3c). The addition of VEGF to the cultures resulted in suppression of *ALP* gene expression in CoD (Fig. 3b), but in the CoE there was no effect on *ALP* expression apart from one patient sample, in which it was significantly reduced (Fig. 3d).

### Type 1 collagen, VEGF and vWF gene expression in co-cultures of EC and FFDSCs

**Fig. 4:**
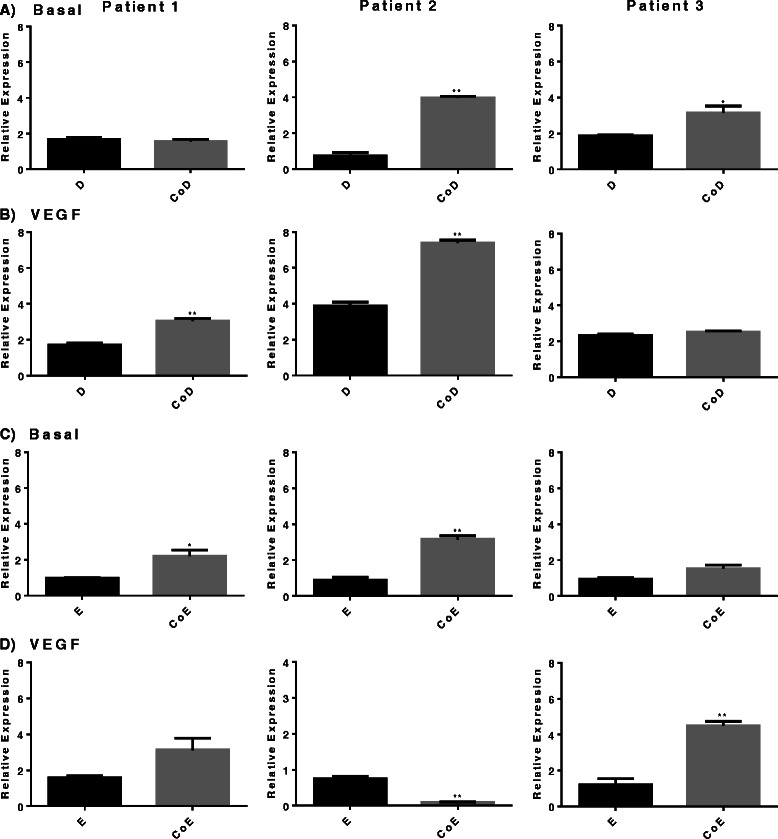
Type I collagen relative gene expression. Comparison in mono-/co-cultures supplemented with or without vascular endothelial growth factor (*VEGF*) in three patient samples. **a** Basal diaphyseal/human umbilical vein endothelial cell (HUVEC) mono-/co-cultures; **b** diaphyseal/HUVEC mono-/co-cultures supplemented with VEGF; **c** basal epiphyseal/HUVEC mono-/co-cultures; **d** Epiphyseal/HUVEC mono-/co-cultures supplemented with VEGF. Results are expressed as mean ± SD: *p ≤0.05, **p ≤0.01, ***p ≤0.001. *CoD* diaphyseal co-culture, *CoE* epiphyseal co-culture

*Type I collagen* expression was significantly increased predominantly in basal diaphyseal co-cultures in contrast to monoculture groups (Fig. 4a). Similarly, in CoD treated with VEGF, a significant increase in *type I collagen* was observed (Fig. 4b). Basal CoE also showed a significant increase in *type I collagen* expression in two of three samples (Fig. 4c). In contrast, the addition of VEGF produced a variable response in the three patient samples analysed (Fig. 4d). Analysis of relative gene expression of *von Willebrand factor* (*vWF*) (Additional file 1) and *VEGF* indicated variable expression of both in response to co-culture conditions in basal media and to the addition of VEGF (Additional file 2). Responses to VEGF in co-cultures of both CoD and CoE were not significant. No significant changes in *VEGF* gene expression in mono- or co-cultures with or without additional VEGF were observed.

### VEGF-receptor FLT-1 and KDR gene expression in co-cultures of EC and FFDSCs 

**Fig. 5:**
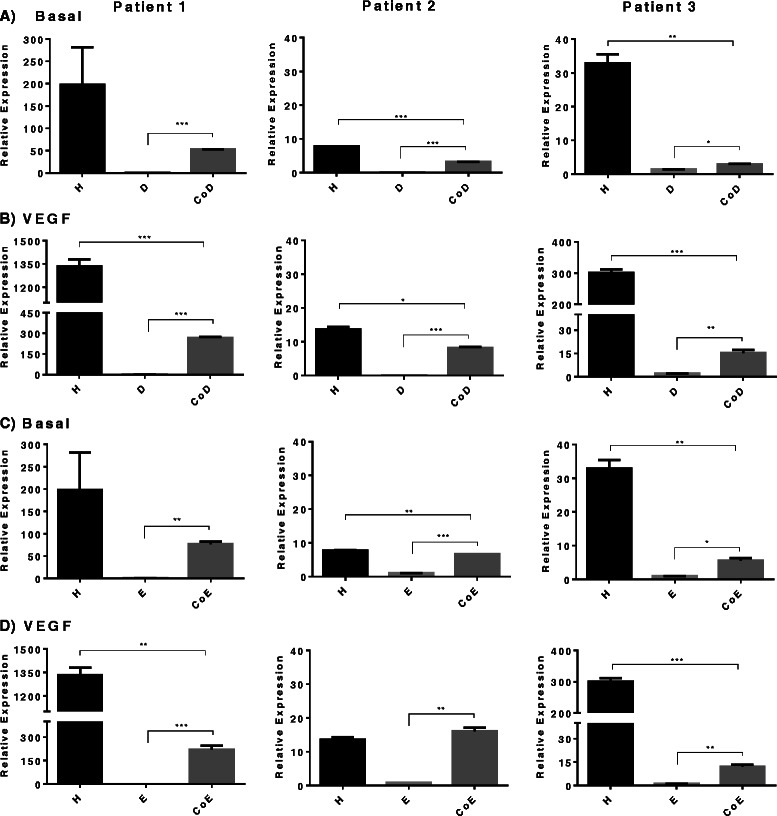
Fms-related-tyrosine kinase 1 (FLT-1)/vascular endothelial growth factor (*VEGF*)-R1 relative gene expression. Comparison in mono-/co-cultures supplemented with or without VEGF in three patient samples. **a** Basal diaphyseal/human umbilical vein endothelial cell (HUVEC) mono-/co-cultures; **b** diaphyseal/HUVEC mono-/co-cultures supplemented with VEGF; **c** basal epiphyseal/HUVEC mono-/co-cultures; **d** epiphyseal/HUVEC mono-/co-cultures supplemented with VEGF. Results are expressed as mean ± SD: *p ≤0.05, **p ≤0.01, ***p ≤0.001. *CoD* diaphyseal co-culture, *CoE* epiphyseal co-culture

The addition of VEGF to HUVEC cultures significantly increased expression levels of *FLT-1 (R1)*. In contrast in basal media of both monocultures D and E *FLT-1 (R1)* expression was found to be extremely low (Fig. 5a, c), which was not further enhanced with VEGF supplementation to the monocultures (Fig. 5b/d). However, in the co-cultures, significant increase in receptor expression (D/CoD = maximum approximately 80-fold; E/CoE = maximum approximately 116-fold) in basal and approximately 2-fold to 3-fold further increase with the addition of VEGF in both CoD and CoE, compared to their respective co-cultures in basal medium was observed. Overall, the mRNA levels of *FLT-1 (R1)* in co-cultures displayed a greater increase when VEGF was added to the media compared to cultures in basal media; however, sample variability precluded any conclusion as to whether CoD or CoE displayed a greater response.

**Fig. 6:**
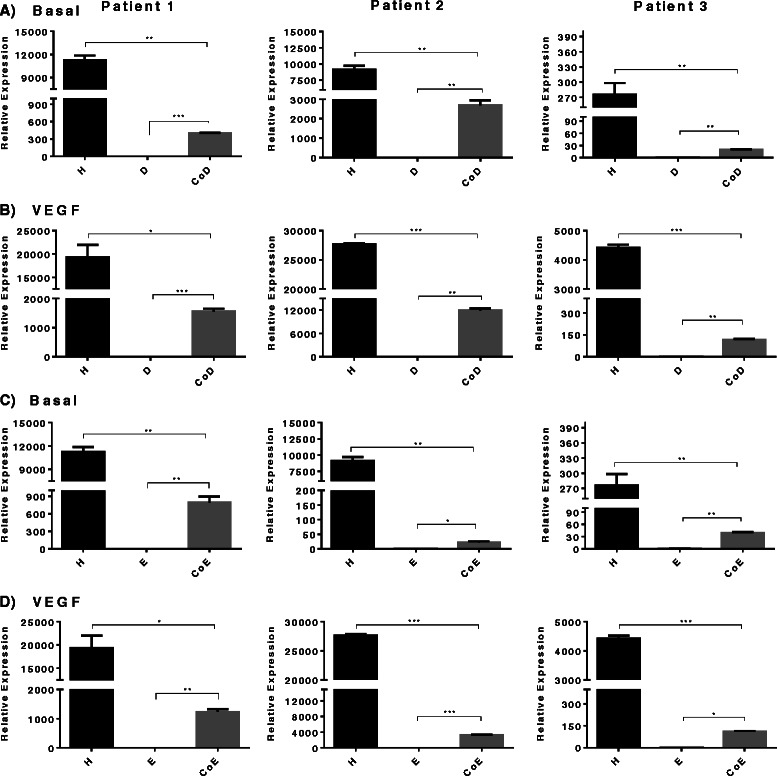
Kinase-insert domain receptor (KDR)/vascular endothelial growth factor (VEGF)-R2 relative gene expression. Comparison in mono-/co-cultures supplemented with or without VEGF in three patient samples. **a** Basal diaphyseal/ human umbilical vein endothelial (HUVEC) mono-/co-cultures; **b** diaphyseal/HUVEC mono-/co-cultures supplemented with VEGF; **c** basal epiphyseal/HUVEC mono-/co-cultures; **d** epiphyseal/HUVEC mono-/co-cultures supplemented with VEGF. Results are expressed as mean ± SD: *p ≤0.05, **p ≤0.01, ***p ≤0.001. *CoD* diaphyseal co-culture, *CoE* epiphyseal co-culture

The levels of *KDR (R2)* gene expression in basal HUVEC was significantly elevated (2-fold to 15-fold increase) when cultured with VEGF (Fig. 6b, d). In contrast to HUVEC expression of the *KDR (R2)* gene, negligible receptor expression was observed in the D and E monocultures (Fig. 6a–d). However, in the co-cultures we found that receptor expression compared to the D and E monocultures were elevated with a 4-fold to 6-fold increase in CoD and a 1.5-fold to 116-fold increase in CoE, respectively, with the addition of VEGF to the cultures (Fig. 6b, d).

### The osteogenic effects of VEGF on organotypic embryonic E11 chick femur cultures

**Fig. 7:**
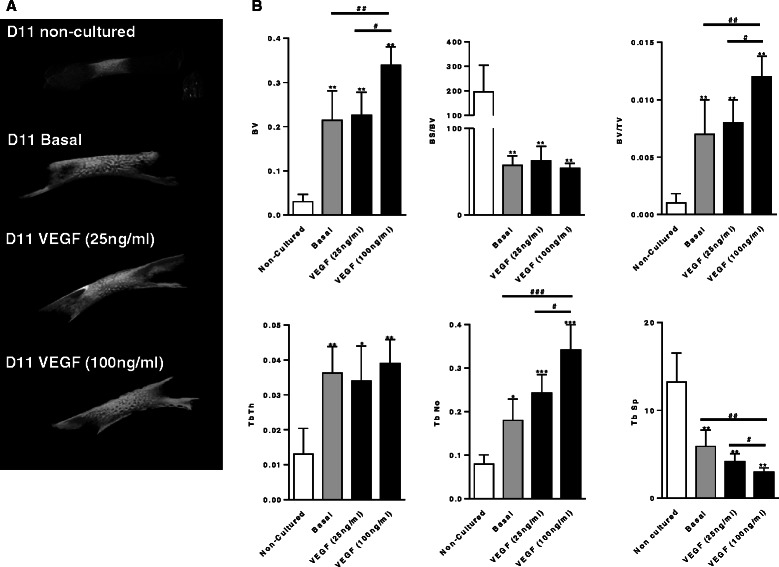
The effect of vascular endothelial growth factor (*VEGF*) (25 ng/ml and 100 ng/ml) on organotypic cultures of embryonic femurs (*E11*). **a** Micro computed tomography images. **b** Micro computed tomography analysis of bone volume (*BV*), bone surface/bone volume (*BS/BV*), bone volume/tissue volume (*BV/TV*), trabecular thickness (*Tb.Th*), trabecular number (*Tb.No*) and trabecular spacing (*Tb.Sp*) (n = 4 femurs per group). Significant differences compared to non-cultured femurs, *p ≤0.05, **p ≤0.01, ***p ≤0.001. Significant difference between basal and VEGF cultured femurs represent mean ± SD: #*p* ≤0.05, ^##^ *p* ≤0.01, ^###^ *p* ≤0.001

In organotypic chick femur cultures supplementation of VEGF (25 ng/ml) displayed negligible osteogenesis compared to basal cultured femurs. In contrast, addition of VEGF at 100 ng/ml, significantly elevated the osteogenic effect on organotypic cultures of D11 embryonic chick femurs after 10 days in culture (Fig. 7a, b), evidenced by the increase in structural bone parameters (BV, Tb.Th and Tb.No and reduced Tb.Sp) compared to basal cultured femurs.

**Fig. 8:**
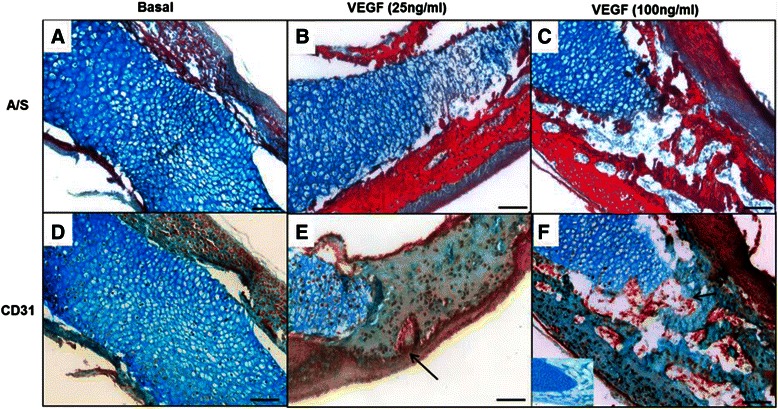
Histological analysis of the effect of vascular endothelial growth factor (*VEGF*) (25 ng/ml and 100 ng/ml) on organotypic cultures of embryonic femurs (*E11*). **a**–**c** Alcian blue/Sirius red staining; **d**–**f** CD31 immunohistochemical staining. CD31-positive cells within the diaphyseal trabecular bone indicated by *arrows*, negative control inset) (n = 4 femurs per group). *Scale bar* 100 μm

Histological analysis of the organotypic chick femur cultures depicted similar outcomes to the μCT results. The mid-diaphyseal region of the control basal femur group was predominantly composed of cartilage (evidenced by Alcian blue staining) with discrete collagenous bone matrix (evidenced by Sirius red staining) in the periosteal regions. In contrast, the collagen staining observed was much denser in the VEGF groups with concomitant reduction of the cartilage region. Interestingly, in the VEGF (100 ng/ml) group, the whole of the mid-diaphyseal region appeared to be made up of trabecular-like bone (Sirius red) with no evidence of cartilage staining, apart from the peripheral, metaphyseal regions (Fig. 8a–c). Furthermore, the addition of VEGF to the organotypic culture of embryonic chick femurs at both concentrations elevated the numbers of CD31-positive cells in the diaphyseal region of the femur compared to the basal cultured femurs (Fig. 8d–f).

## Discussion

The current study has examined the interactive processes of the three major cell types (diaphyseal/epiphyseal femur cells and endothelial cells) present during early foetal femoral development to further understand the concomitant angiogenesis and osteogenesis that occurs in skeletal development, and is recapitulated in fracture repair. Initially, we demonstrated that the early osteogenic differentiation marker ALP was elevated when foetal femur-derived stem cells (FFDSC) from both the diaphyseal and epiphyseal regions were co-cultured in direct contact with endothelial cells. This corresponded to previous reports that adult osteoprogenitor cells in direct culture with HUVECs induce an increase in ALP [9, 11].

Interestingly, in our studies, we found that the supplementation of the potent angiogenic growth factor VEGF to these co-cultures resulted in differing responses between the diaphyseal and epiphyseal FFDSCs. VEGF supplementation reduced the levels of ALP in the diaphyseal/HUVEC co-culture group, whereas in contrast, there was a variable response in the epiphyseal/HUVEC group compared to both co-culture groups without VEGF supplementation. Within the co-cultures, an increase in the gene expression of *type 1 collagen* compared to monocultures was observed, however, the addition of VEGF had a negligible effect. Similarly, there was no increase in the gene expression of *VEGF* and *vWF* in the co-cultures. Upon analysis of the VEGF receptor genes 1 *(FLT-1)* and 2 *(KDR)*, we found significant differences in the co-cultures with further increases in the receptor expression upon addition of VEGF, with receptor 2 being more sensitive to VEGF stimulation compared to receptor 1.

Within the skeletal environment, vascular cells play a fundamental role in modulating the osteogenic progression of the developing bone and in the temporal cascade of bone defect repair. Human foetal skeletal populations contain primitive progenitor/bone stem cell populations, which maintain a high degree of proliferation and plasticity potential [32]. The cells from the epiphyseal and diaphyseal regions of the foetal femur provide a predetermined chondrogenic and osteogenic population of cells respectively, hence, being ideal for investigations into skeletogenesis [33, 34]. We hypothesised that co-integration of vascular endothelial cells with these multipotent foetal femur cells could modulate osteogenesis and to a degree, angiogenesis, in line with endochondral bone formation during bone development and repair.

The response of the cells in contact co-cultures of early human skeletal and endothelial cells appear to be dependent on the differentiation state of the cells present in culture. Although the diaphyseal and epiphyseal cells are predominantly associated with osteogenic and chondrogenic phenotypes, respectively, these populations are not homogenous. While the diaphyseal and epiphyseal cells are of an immature phenotype and of limited development, the populations represent a developmental continuum with immature osteoprogenitors and chondroprogenitors, that also contains mature chondrocyte sub-populations. This was reflected in the variability and fluctuation of expression observed in osteogenic markers ALP and *type I collagen* and the lack of change in angiogenic factors *vWF* and *VEGF* across the differing patient samples within the study. Additionally, it is important to note, patient-to-patient variation will contribute to the variability in the results observed (indicated by the observed differences in ALP activity and *ALP* gene expression) and that gene expression and protein expression are not always correlated. This variation was also reflected in increased levels of ALP in one patient, displaying an enhanced osteogenic phenotype. It would appear that cells isolated from these femoral regions at this stage of differentiation are not responsive to the angiogenic stimulation of VEGF, evidenced by low levels of mRNA. Low *VEGF* mRNA expression may additionally reflect modulation through its cognate receptors on surrounding cells, as reviewed by Marini et al. [35]. It is possible that the cells themselves may produce and release VEGF into the media, resulting in variability of expression; however, Grellier et. al. previously measured VEGF release by osteoprogenitor cells and HUVECs in co- and mono-cultures but only detected the VEGF in the supernatant of osteoprogenitors after 48 h. Similarly, Leszczynska et al. examined VEGF release by HUVECs and human bone marrow-derived cells in various co-culture ratios over 4 and 7 days. The authors noted significant amounts of VEGF in the human bone marrow cells. Thus, given the relatively high concentration of VEGF (100 ng/ml) in the current study, the possible paracrine release by the cells would have contributed only minimally to the overall effect observed [12, 36].

Ramasamy et al. have postulated that bone formation is governed by a specialised angiogenic mode, implicating the Notch signalling pathway as the angiogenic pathway acting directly on osteoblasts. Disruption of Notch signalling resulted in impaired blood vessel formation, reduced bone formation and chondrocyte differentiation. Furthermore, Noggin (BMP-antagonist) restored these vascular and skeletal disruptions [37]. In addition, the authors identified endothelial cells with high expression of Endomucin (Emcn) and CD31 residing in the distal arches of metaphyseal vessels of long bones, with highly expressed VEGF receptors 1, 2 and 3 in the bone.

Hellstrom et al., demonstrated that distinct types of endothelial cells are involved in angiogenesis and can modulate their phenotype according to the stages of neovascularisation and VEGF availability. De-activation and activation of the Dll-4 (Delta-like-ligand 4) and Notch 1 pathway, respectively, was observed to switch the differential behaviour of the endothelial cells on and off resulting in a highly effective mechanism in functional and optimised blood vessel formation [38]. As previously established, the exposure and sequestration of VEGF is in turn orchestrated mainly by VEGF receptors 1 and 2, and similar to our findings any differentiation and change in phenotype of the ECs cells can have an effect on how these cells respond to contact with osteoblastic cells and/or exogenous VEGF.

Osteoblasts express VEGF receptors [39, 40], and contact with HUVECs in co-culture may trigger the inhibition of VEGF receptors in order to prepare the tissue for bone healing and possibly inhibit bone resorption at this differential stage, which would be enhanced by VEGF [25, 40, 41]. In HUVEC monocultures, high expression levels of *KDR (R2)* and *FLT-1 (R1)* were observed indicating the ECs were in a highly proliferative state [42], which in turn was increased by the addition of VEGF to the HUVECs. In contrast, HUVECs in contact with osteoblastic cells in basal media, displayed reduced proliferation in preparation for differentiation towards mature ECs. However, the addition of VEGF switched the balance back again from differentiation towards proliferation, increasing VEGF receptor mRNA. Recent work has shown the importance of endothelial cells ability to differentiate into skeletal cells by endothelial-mesenchymal transition [43], and their subsequent role in heterotopic ossification. However, it cannot be dismissed that mesenchymal skeletal progenitor cells may have the capacity to differentiate into endothelial cells as evidenced by studies demonstrating Rho/MRTF-A signalling pathway as a critical factor in VEGF differentiation of mesenchymal stem cells (MSC) to endothelial cells and that the combined effect of matrix elasticity and VEGF can also modulate MSC to the endothelial phenotype, respectively [44, 45]. Analysis of gene expression of the relevant osteogenic and angiogenic markers in each of the separate HUVEC and foetal skeletal cell populations will indicate the modulating effects occurring in this co-culture system. However, as this is a contact bound co-culture effect, we were concerned that mechanical separation of the individual cell types would disrupt the very connections under analysis in the contact co-cultures. Elegant studies by Grellier et al*.* separated the HUVEC portion from their co-culture system using magnetic cell sorting, however techniques such as magnetic-activated cell sorting (MACS) and fluorescence-activated cell sorting (FACS) typically are not 100 % efficient, therefore caution is required in evaluating data from contact-co-culture isolated cells [36]. Furthermore, we have not examined cell separation from our co-cultures, as foetal femur cells derived from foetal femoral tissue are not a homogenous population of cells (chondrogenic, osteogenic and endothelial populations as well as skeletal and endothelial progenitor and stem sub-populations) and thus, a concern was the inability to generate 100 % verifiable efficient separation between our cell types.

In an organotypic cultured femur model, we found that embryonic chick femurs at E11 displayed significant levels of new bone formation when supplemented with a high dose of VEGF. No expression of the endothelial cell marker CD31 was observed in non-cultured femurs (data not shown), and in basal cultured femurs, positive CD31 cells were found to be residing in the periosteal region of the femur. In marked contrast, VEGF addition resulted in an increase in CD31-positive cells in the mid-diaphyseal region of these cultured femurs, with migration into the newly formed trabecular bone cavities, which correlated with an increase in osteogenesis. In bone development VEGF is highly expressed in the hypertrophic chondrocytes and inhibition of VEGF by administering a soluble chimeric VEGF receptor, significantly reduces blood vessel invasion into the hypertrophic region of long bones resulting in expansion of the growth plate and a reduction in trabecular bone formation in 24-day-old mice [20]. The current results indicate that at the early stages of the osteogenic process in the femur, VEGF plays a critical part in the developing femur with the potential migration/differentiation of endothelial phenotype cells in the mid-diaphyseal region of bone. Interestingly, the location of these progenitor cells undergoing differentiation due to VEGF, is unclear. Thus, fate tracing of these population of cells (for example, whether or not they are of pericyte origin), will inform additional approaches to recruit these specialised cells for future therapies to regenerate bone. These studies confirm the importance of direct cell contact as a crucial prerequisite in early osteogenesis. Further modulation of the co-culture system to deliver a facile temporal response to enable osteogenesis and angiogenesis and our understanding of the molecular interaction of vascular cells and associated osteoprogenitors will be crucial for future bone regenerative therapies.

## Conclusion

This study has demonstrated that contact co-cultures of foetal femur-derived stem cells and human umbilical vein endothelial cells enhance mechanisms of osteogenesis and angiogenesis in bone, evident in a significant increase in early osteogenic markers ALP and *Col1* and a significant modulation of *VEGF receptor* activity in co-cultures that did not result in modulation of *VEGF* gene expression. We observed intricate differential responses from cells originating from the diaphysis and epiphysis of the foetal femur. Diaphyseal co-cultures triggered a stronger response of *Col1* gene expression with and without addition of VEGF than the epiphyseal fractions; however epiphyseal co-cultures displayed greater ALP activity compared to diaphyseal co-culture groups. Supplementation of VEGF decreased the enhancing effect of co-cultures in the diaphyseal co-culture group in vitro; however, in an ex vivo model of chick femoral defect, a significant increase in bone formation with the addition of VEGF and stimulation of CD31-positive cells within the primary ossification centre, was demonstrated. This study shows great future therapeutic potential in using co-culture models for fracture repair.

## Additional files

Additional file 1:
**von Willebrand factor relative gene expression.** Comparison in mono-/co-cultures supplemented with or without vascular endothelial growth factor (*VEGF*) in three patient samples. **a** Basal diaphyseal/human umbilical vein endothelial cell (HUVEC) mono-/co-cultures; **b** diaphyseal/HUVEC mono-/co-cultures supplemented with VEGF; **c** basal epiphyseal/HUVEC mono-/co-cultures; **d** epiphyseal/HUVEC mono-/co-cultures supplemented with VEGF. Results are expressed as mean ± SD: **p* ≤0.05, ***p* ≤0.01, ****p* ≤0.001. (PDF 16 kb) Additional file 2:
**Vascular endothelial growth factor** **(** ***VEGF*** **) relative gene expression** **.** Comparison in mono-/co-cultures supplemented with or without VEGF in three patient samples. **a** Basal diaphyseal/human umbilical vein endothelial cell (HUVEC) mono-/co-cultures; **b** diaphyseal/HUVEC mono-/co-cultures supplemented with VEGF; **c** basal epiphyseal/HUVEC mono-/co-cultures; **d** epiphyseal/HUVEC mono-/co-cultures supplemented with VEGF. Results are expressed as mean ± SD: **p* ≤0.05, ***p* ≤0.01, ****p* ≤0.001; n = 3. (PDF 16 kb)

## Abbreviations

ALP: alkaline phosphatase
α-MEM: minimal essential medium, α-modification
ANOVA: analysis of variance
BMP: bone morphogenic protein
BSA: bovine serum albumin
BS/BV: bone surface/volume ratio
CD31: cluster of differentiation 31 - PECAM 1
CoD: diaphyseal co-culture
CoE: epiphyseal co-culture
Ct: cycle threshold
D: diaphyseal
E: epiphyseal
EC: endothelial cell
ECGS: endothelial cell growth supplement
FCS: foetal calf serum
FFDSC: foetal femur-derived stem cell
FLT-1: Fms-related-tyrosine kinase 1 (vascular endothelial growth factor receptor 1)
HBMC: human bone marrow cell
HBMSC: human bone marrow stromal cells
HUVEC: human umbilical vein endothelial cell
kDa: kiloDalton
KDR: kinase-insert domain receptor (vascular endothelial growth factor receptor 2)
MSC: mesenchymal stem cells
PBS: phosphate-buffered saline
Pen/Strep: penicillin/streptomycin
PFA: paraformaldehyde
pNPP: P-nitrophenol phosphate
Tb.N: trabecular number
Tb.Sp: trabecular spacing
Tb.Th: trabecular thickness
TCM: tissue culture medium
μCT: micro-computed tomography
VEGF: vascular endothelial growth factor
vWF: von Willebrand factor
