# Supplementary figures and images for: Human endothelial and foetal femur-derived stem cell co-cultures modulate osteogenesis and angiogenesis
Source: Stem Cell Res Ther. 2016 Jan 18;7:13. doi: 10.1186/s13287-015-0270-3 (PMC4717648; doi:10.1186/s13287-015-0270-3)

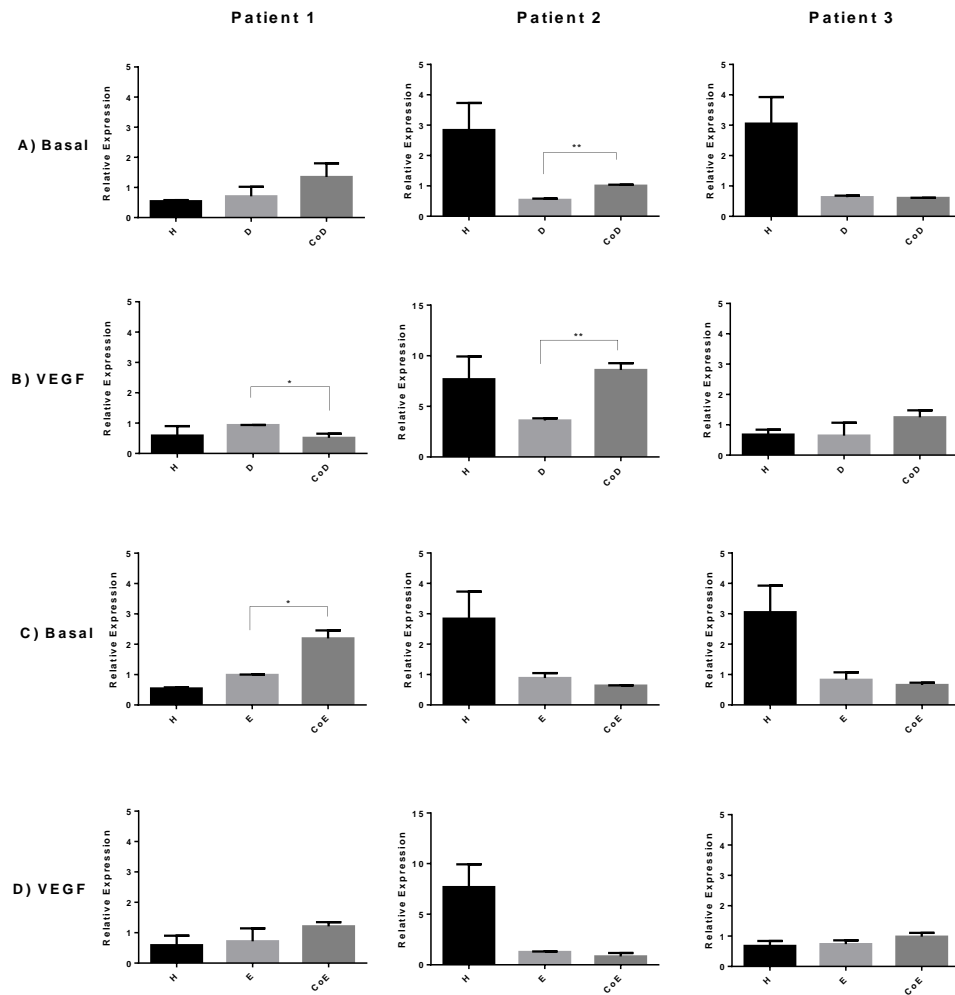

Additional file 1

Supplement: Additional file 1: — von Willebrand factor relative gene expression. Comparison in mono-/co-cultures supplemented with or without vascular endothelial growth factor (VEGF) in three patient samples. a Basal diaphyseal/human umbilical vein endothelial cell (HUVEC) mono-/co-cultures; b diaphyseal/HUVEC mono-/co-cultures supplemented with VEGF; c basal epiphyseal/HUVEC mono-/co-cultures; d epiphyseal/HUVEC mono-/co-cultures supplemented with VEGF. Results are expressed as mean ± SD: *p ≤0.05, **p ≤0.01, ***p ≤0.001. (PDF 16 kb) [file 13287_2015_270_MOESM1_ESM.pdf]

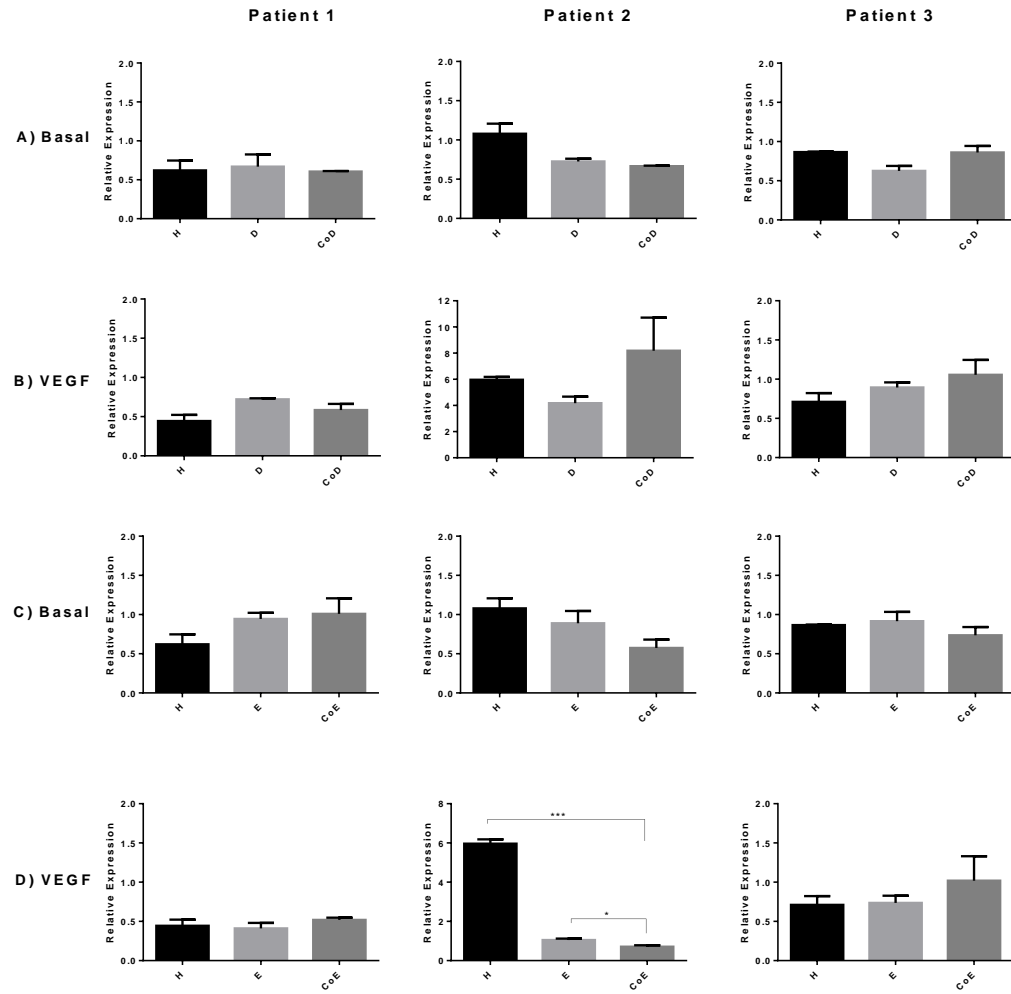

Additional file 2

Supplement: Additional file 2: — Vascular endothelial growth factor ( VEGF ) relative gene expression . Comparison in mono-/co-cultures supplemented with or without VEGF in three patient samples. a Basal diaphyseal/human umbilical vein endothelial cell (HUVEC) mono-/co-cultures; b diaphyseal/HUVEC mono-/co-cultures supplemented with VEGF; c basal epiphyseal/HUVEC mono-/co-cultures; d epiphyseal/HUVEC mono-/co-cultures supplemented with VEGF. Results are expressed as mean ± SD: *p ≤0.05, **p ≤0.01, ***p ≤0.001; n = 3. (PDF 16 kb) [file 13287_2015_270_MOESM2_ESM.pdf]
